# Acute abducens nerve palsy following coronavirus disease 2019 vaccination: a case report

**DOI:** 10.1186/s13256-024-04681-2

**Published:** 2024-08-06

**Authors:** Chen-Ting Wang, Jie-Yuan Li

**Affiliations:** Department of Neurology, E-DA Hospital, I-Shou University, Kaohsiung, Taiwan

**Keywords:** Abducens, COVID-19, Vaccination

## Abstract

**Background:**

Abducens nerve palsy is the most common isolated ocular cranial nerve palsy. In adults, nontraumatic etiologies of isolated sixth cranial nerve palsy can include vascular disease, inflammation, tumors, and a prior history of infection.

**Case presentation:**

We present a case of a 52-year-old Asian male who developed acute abducens nerve palsy after vaccination with the AstraZeneca coronavirus disease 2019 vaccine. A complete workup including magnetic resonance imaging of the brain and orbits revealed no abnormalities. The patient experienced a gradual recovery over 10 weeks through alternative eye patching. The abducens nerve palsy is postulated to be correlated with the coronavirus disease 2019 vaccine.

**Conclusion:**

Despite the recognized efficacy and cost benefits of coronavirus disease 2019 vaccines, clinicians should be aware of the possible association between cranial nerve palsies and coronavirus disease 2019 vaccines.

## Background

Sixth cranial nerve palsy, also known as abducens nerve palsy, refers to the dysfunction of the abducens nerve. It is the most common isolated ocular cranial nerve palsy [1]. In adults, nontraumatic etiologies of isolated sixth cranial nerve palsy can include vascular disease, inflammation, tumors, and previous infections [2].

Here, we present a case of a 52-year-old male who developed acute abducens nerve palsy after vaccination with the AstraZeneca coronavirus disease 2019 (COVID-19) vaccine.

## Case presentation

A 52-year-old Asian male, with no underlying health conditions, presented to the neurology outpatient department with acute binocular diplopia, 12 days after receiving the AstraZeneca COVID-19 vaccine. At the time of the visit, intermittent pain over the left eye was also noted. The patient’s vital signs were within the normal range, with no fever detected.

**Fig. 1:**
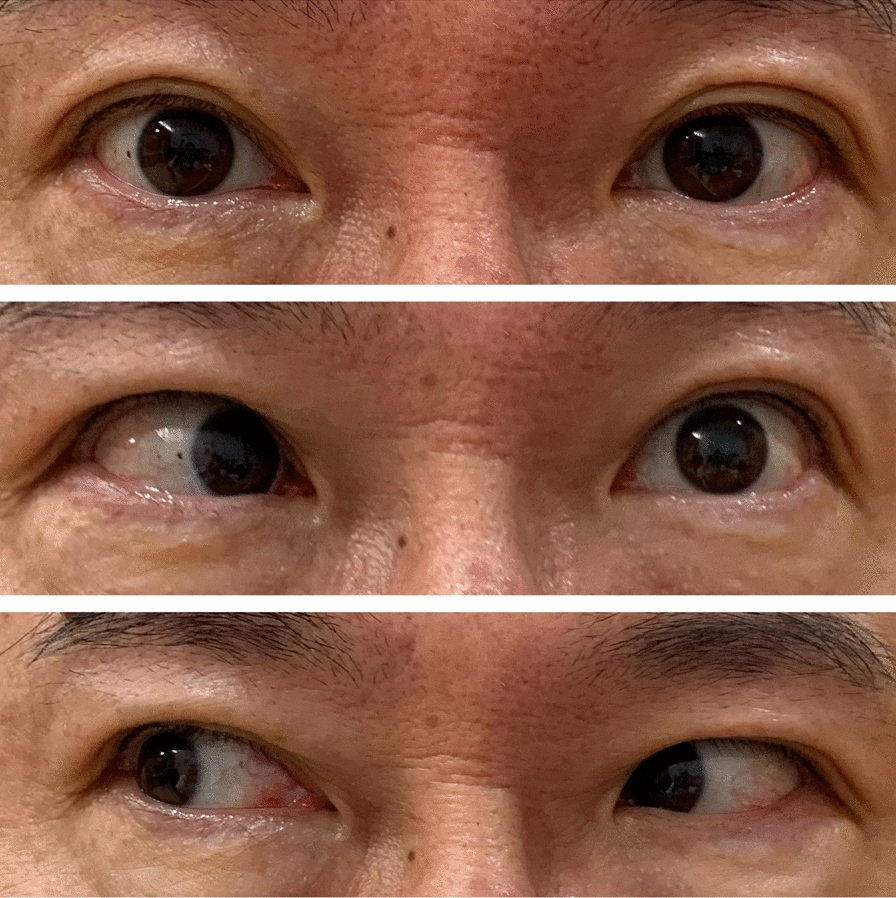
The neurological examination revealed primary estropia of the left eye and an obvious abduction deficit of the left eye on left gaze

Neurological examination revealed primary esotropia of the left eye and an obvious abduction deficit of the left eye on the left gaze. The patient noted that the binocular diplopia was alleviated with right gaze. There was no afferent pupillary defect, and visual acuity was intact. Further neurological examination revealed that other cranial nerves were spared. Therefore, the patient was diagnosed with isolated sixth cranial nerve palsy (Fig. 1).

The patient underwent blood tests, including a complete blood test, metabolic profiles including blood sugar and hemoglobin A1c (HbA1c), D-dimer test, erythrocyte sedimentation rate (ESR), and C-reactive protein (CRP). These blood tests were all normal. Magnetic resonance imaging (MRI) of the brain and orbits with and without gadolinium showed no abnormal signal, enhancement, or mass lesion.

Based on the above findings, acute abducens nerve palsy was postulated to be correlated with the COVID-19 vaccine. We asked the patient to wear an alternative eye patch a few hours daily to prevent lazy eye. He experienced a gradual recovery over 10 weeks at the outpatient department follow-up.

## Discussion

Vaccination-induced abducens nerve palsy is generally diagnosed after ruling out other causes. In this patient, traumatic etiology and the presence of a brain tumor were excluded based on previous medical history and brain imaging. Patients with vascular risk factors such as diabetes mellitus and hypertension may have isolated abducens nerve palsy. Our patient had no underlying disease before, and his measured blood pressure and laboratory data, including blood sugar and HbA1c, were within the normal range. Therefore, microvascular mononeuropathy was less likely.

Isolated sixth cranial nerve palsy has been reported in patients with COVID-19 infection, along with other presenting symptoms such as fever, sore throat, ageusia, and anosmia [3]. In our case, these associated symptoms did not occur throughout the entire course of the patient’s condition, and the COVID-19 screening test was negative. Therefore, COVID-19 infection was unlikely to be the cause of the abducens nerve palsy.

A review of cranial nerve palsies occurring after routine immunization was first discussed based on results from the US Vaccine Adverse Event Reporting System (VAERS) [4]. The cranial nerves most commonly involved in these patients were the oculomotor, trochlear, and abducens. The vaccines listed on reports of cranial nerve palsies included the seasonal trivalent inactivated influenza vaccine, *Hemophilus influenzae* type b vaccine and human papillomavirus vaccine quadrivalent, as well as the live measles, mumps, and rubella vaccine.

The mechanism behind the pathogenesis of vaccine-induced abducens nerve palsy remains unclear. It has been suggested that injury to the abducens nerve occurs due to a neurotropic effect of the infectious agent, demyelination from an immune-mediated reaction, and localized arteritis or microinfarction of the abducens nerve [5]. Reyes-Capo *et al*. reported a case of acute abducens nerve palsy in a healthy 59-year-old female 2 days after receiving the Pfizer–BioNTech COVID-19 vaccine [6]. The authors hypothesized that the patient developed a viral-like inflammatory reaction to the vaccine, inciting an immune-mediated indirect insult along the abducens nerve. Neelam-Pawer *et al*. also reported a case of left abducens nerve palsy in a 23-year-old male after receiving the Covishield COVID-19 vaccine [7]. It is suggested that the genetic material of the coronavirus is expressed following the administration of a vaccine, which stimulates an immune response similar to postviral infections. In our case, unilateral painful abducens nerve palsy with normal cranial imaging was proposed to be correlated with the etiology of an immune-mediated reaction induced by the COVID-19 vaccine.

The recovery rates of unilateral sixth nerve palsy were investigated in patients with diplopia excluding traumatic etiology [8]. The results showed that 78.4% of patients underwent spontaneous recovery, and 84.4% recovered within 4 months. Similarly, our patient recovered fully by 10 weeks post-onset of isolated abducens nerve palsy.

## Conclusion

Although the efficacy and cost benefits of COVID-19 vaccines are widely accepted, neurological complications may occur even in otherwise healthy individuals. Clinicians should consider the possible correlation between cranial nerves palsies and COVID-19 vaccines to ensure appropriate treatment strategies.

## Data Availability

The authors confirm that the data supporting the findings of this study are available within the article and its supplementary materials.

## Abbreviations

COVID-19: Coronavirus disease 2019
ESR: Erythrocyte sedimentation rate
CRP: C-reactive protein
MRI: Magnetic resonance imaging
